# Effect of anxiety and depression on self-reported adverse reactions to COVID-19 vaccine: a cross-sectional study in Shanghai, China

**DOI:** 10.1186/s12889-023-15118-8

**Published:** 2023-03-03

**Authors:** Zhitong Zhou, Junwei Shen, Miaomiao Zhao, Xiaoying Zhang, Tao Wang, Jue Li, Xudong Zhao

**Affiliations:** 1 Clinical Research Center for Mental Disorders, Shanghai Pudong New Area Mental Health Center, School of Medicine, Tongji University, Shanghai 200124, China; 2 Institute of Clinical Epidemiology and Evidence-Based Medicine, School of Medicine, Tongji University, Shanghai 200092, China; 3 School of Clinical Medicine, Shanghai University of Medicine and Health Sciences, Shanghai 201318, China; 4 College of public Health, Shanghai University of Medicine and Health Sciences, Shanghai 201318, China

**Keywords:** Anxiety, Depression, COVID-19, Vaccine, Adverse reactions

## Abstract

**Background:**

The association of anxiety and depression with adverse reactions after receipt of coronavirus disease 2019 (COVID-19) vaccine is not clear among the general population. This study aims to evaluate the effect of anxiety and depression on self-reported adverse reactions to COVID-19 vaccine.

**Methods:**

The cross-sectional study was conducted during April–July 2021. Participants completing the two doses of vaccine were included in this study. Sociodemographic information, anxiety and depression levels and adverse reactions after the first dose of vaccine for all participants were collected. The anxiety and depression levels were assessed by the Seven-item Generalized Anxiety Disorder Scale and the Nine-item Patient Health Questionnaire Scale, respectively. The multivariate logistic regression analysis was used to examine the association between anxiety and depression and adverse reactions.

**Results:**

A total of 2161 participants were enrolled in this study. The prevalence of anxiety and depression was 13% (95% confidence interval (CI), 11.3–14.2%) and 15% (95%CI, 13.6–16.7%), respectively. Of the 2161 participants, 1607 (74%; 95% CI, 73–76%) reported at least one adverse reaction after the first dose of the vaccine. Pain at the injection site (55%) and fatigue and headache (53% and 18%, respectively) were the most commonly reported local and systemic adverse reactions, respectively. Participants with anxiety or depression or both were more likely to report local and systemic adverse reactions (*P* < 0.05).

**Conclusion:**

The results suggest that anxiety and depression increase the risk of self-reported adverse reactions to COVID-19 vaccine. Consequently, appropriate psychological interventions before vaccination will help to reduce or alleviate symptoms of vaccination.

**Supplementary Information:**

The online version contains supplementary material available at 10.1186/s12889-023-15118-8.

## Introduction

Globally, the coronavirus disease 2019 (COVID-19) pandemic has caused more than 300 million infections and more than 5 million deaths as of January 2022 [1], with a serious impact on global health and economy. Moreover, the pandemic is still developing. Although wearing masks and maintaining social distancing can mitigate the spread of the virus, vaccination is the most effective measure to control epidemic development [2]. When population immunity reaches 67%, COVID-19 infections are expected to decrease [3]. Currently, more than 100 candidate vaccines are in clinical trials and several vaccines in multiple countries have been approved [4]. Globally, more than 10 billion doses of COVID-19 vaccines have been administered [5] and 65.5% of the world population has received at least one dose of a COVID-19 vaccine [6], but many people remain unvaccinated. Among these people, the safety and effectiveness of vaccines can be an important factor affecting their vaccination [7–9], which does not help halt the transmission of the virus.

COVID-19 vaccine can effectively reduce the incidence of infections, serious adverse events and deaths caused by severe acute respiratory syndrome coronavirus 2 (SARS-CoV-2) [10]. The effectiveness of COVID-19 vaccination is generally between 50% and 95% according to the reported data from phase III clinical trials [11]. The main local and systemic adverse reactions of COVID-19 vaccine are mild or moderate in severity [12, 13]. In published clinical trials of COVID-19 vaccines, the overall incidence of solicited adverse reactions after vaccination was 21–29% for inactivated vaccines (BBIBP-CorV and CoronaVac) [12, 14–16], and 64–81% for adenovirus vector vaccines (Ad5 vectored vaccine) in China [17–19]. Additionally, the incidence of solicited local adverse events was 84–89%, solicited systemic adverse events was 55–79% and serious adverse events was 0.6% after vaccination for the mRNA-1273 vaccine in the US [20]. A multinational clinical trial, including the United States, Argentina, Brazil, South Africa, Germany and Turkey, showed that the most commonly reported local adverse reactions was pain at the injection site (66–83%) and the most common local reported systemic adverse reactions were fatigue (34–59%) and headache (25–52%) for mRNA vaccine (BNT162b2) [21]. A clinical trial in Australia reported solicited adverse events of 90%, vaccine-related unsolicited adverse events of 13% and unsolicited adverse events of 36% for MF59-adjuvanted spike glycoprotein-clamp vaccine [22].

As the global pandemic of COVID-19, the psychological burden of the population is increasing. Globally, the pandemic results in a 27·6% increase in cases of major depressive disorders and 25·6% increase in cases of anxiety disorders [23]. A nationally representative US study (336,525 participants) indicated that adults in April and May 2020 were more than three times likely to suffer from depression, anxiety or both compared to the first half of 2019 [24]. A cross-sectional study of 7236 volunteers in China found that the prevalence of generalized anxiety disorder and depression was 35.1% and 20.1%, respectively [25]. During the COVID-19 pandemic, the fear of infecting with COVID-19 and the safety and effectiveness of SARS-CoV-2 vaccine, having not enough surgical masks and food supplies, lower socioeconomic status and social support were associated with poorer psychological status, and sufficient medical resources, up-to-date and accurate information were protective factors of mental health [26–29].

Psychological factors play an important role in the safety and effectiveness of vaccination, and the immune system’s response to vaccine can be impaired by poor psychological status [28, 30]. A randomized controlled trial among people aged 65–74 years showed that the populations with anxiety or depression were more likely to report systemic adverse reactions after an influenza vaccine [31]. Additionally, a cross-sectional study conducted in people aged 65–85 years suggested that depression symptoms were positively associated with severe adverse reactions to COVID-19 vaccine [32]. A cohort study of 908,689 outpatient vaccination in Germany demonstrated that pre-existing anxiety and depression significantly elevated the risk of reported side effects of COVID-19 vaccine [33].

However, the association of anxiety and depression with adverse reactions after receipt of COVID-19 vaccine is not clear among the general population in China. Therefore, we aimed to assess the effect of anxiety and depression on self-reported adverse reactions to COVID-19 vaccine among the general population based on a cross-sectional study in Shanghai, China. We hypothesized that anxiety and depression can increase the risk of self-reported adverse reactions in the general population.

## Methods

### Study designs and participants

This study was carried out during April–July 2021 in Tongji university and Sanlin and Huamu communities. After obtaining participants’ informed consent, participants completing the two doses of COVID-19 vaccine were recruited in this study. Sociodemographic information including sex, age, residential address, working condition and marital status, anxiety and depression levels and adverse reactions after the first dose of vaccine for all participants were collected. All data were collected using Wenjuanxing, an online tool for questionnaire surveys (https://www.wjx.cn/).

### Instruments

The Chinese version of Seven-item Generalized Anxiety Disorder Scale (C-GAD-7) was utilized to assess the anxiety levels of participants [34]. Studies showed that the scale had good reliability and validity [35, 36]. The Cronbach’s α was 0.931 for the C-GAD-7 in this study. Each item scored from 0 to 3 (0 = not at all; 1 = several days; 2 = more than half the days; and 3 = nearly every day), making up total scores with range 0–21: 0–4, normal; 5–9, mild; 10–13, moderate; 14–18, moderately severe; and 19–21, severe [34].

Furthermore, the Chinese version of Nine-item Patient Health Questionnaire Scale (C-PHQ-9) was used to assess the depression levels of participants [37]. Good reliability and validity for the scale were determined in previous studies [38, 39]. The Cronbach’s α was 0.923 of the C-PHQ-9 in this study. Each item scored from 0 to 3 (0 = not at all; 1 = several days; 2 = more than half the days; and 3 = nearly every day), resulting in total scores with a range 0–27. The cut-off of total scores were 0–4 for normal, 5–9 for mild, 10–14 for moderate, 15–19 for moderately severe and 20–27 for severe [40].

### Safety of vaccine

Adverse reactions were termed and graded according to the China State Food and Drug Administration (https://www.nmpa.gov.cn/) (Table S1). In this study, local adverse reactions included pain, redness, swelling and itching at the injection site, and systemic adverse reactions included fever, fatigue, headache, cough, diarrhea, nausea and vomiting.

### Sample size estimation

Reported incidence of adverse reactions of inactivated SARS-CoV-2 vaccine was 29% [12], taking this data as our reference and considering 20% loss to follow-up rate, this study needed 1881 participants.

### Statistical analyses

Categorical variables were presented as frequency and percentage, and the differences between groups were analyzed using χ² test or Fisher’s exact test [41]. The incidence of adverse reactions to vaccine and prevalence of anxiety and depression were calculated as percentages and Clopper–Pearson 95% confidence interval (CI) [21]. The relationships of anxiety and depression with adverse reactions were estimated by multivariate logistic regression analysis. SPSS 29.0 (SPSS Inc., Chicago, IL, USA) and R 4.2.2 were used for all statistical analyses. Two-sided *P*-value < 0.05 was considered statistically significant.

## Results

### Participant characteristics

A total of 2161 participants completing two doses of COVID-19 vaccine during April–July 2021 were included in this study. Of these, 53% were females, 63% were aged < 18–25 years, 57% had undergraduate educational level, 91% lived in urban areas, 67% were not working and 79% were unmarried (Table 1).

**Table 1:** Demographic characteristics of participants

Variables	Overall adverse reactions		*P*-value
	No (n = 554)	Yes (n = 1607)	
Sex (%)			< 0.001
Male	343 (61.9)	669 (41.6)	
Female	211 (38.1)	938 (58.4)	
Age (%)			
< 18–25	343 (61.9)	1037 (64.5)	< 0.001
26–40	133 (24.0)	435 (27.1)	
> 40	78 (14.1)	135 (8.4)	
Educational level (%)			< 0.001
High school and below	68 (12.3)	82 (5.1)	
Undergraduate	315 (56.9)	914 (56.9)	
Master and above	171 (30.9)	611 (38.0)	
Residential address (%)			0.263
Urban	497 (89.7)	1473 (91.7)	
Suburb	24 (4.3)	65 (4.0)	
Countryside	33 (6.0)	69 (4.3)	
Working condition (%)			0.011
Working	182 (32.9)	481 (29.9)	
No working	353 (63.7)	1100 (68.5)	
Retired	19 (3.4)	26 (1.6)	
Marital status (%)			0.023
Unmarried	415 (74.9)	1281 (79.7)	
Married	130 (23.5)	314 (19.5)	
Divorced/widowed	9 (1.6)	12 (0.7)	
Anxiety (%)			< 0.001
Normal	527 (95.1)	1359 (84.6)	
Mild	19 (3.4)	197 (12.3)	
Moderate and above	8 (1.4)	51 (3.2)	
Depression (%)			< 0.001
Normal	520 (93.9)	1315 (81.8)	
Mild	25 (4.5)	222 (13.8)	
Moderate and above	9 (1.6)	70 (4.4)	

Percentages may not be equal to 100 due to rounding.

### Anxiety and depression in the participants

The prevalence of anxiety and depression was 13% (95% CI, 11.3–14.2%) and 15% (95% CI, 13.6–16.7%), respectively, of which mild anxiety and depression were the most common (Figure S1A). Moreover, 207 (10%) participants had both anxiety and depression (Figure S1B).

### Adverse reactions in participants

A total of 1607 (74%; 95% CI, 73–76%) participants reported at least one adverse reaction after the first dose of vaccination. All adverse reactions reported by participants were mild or moderate in severity. The most commonly reported local adverse reaction was pain at the injection site (55%). Fatigue and headache were the most commonly reported systemic adverse reactions (53% and 18%, respectively) (Figure S2A and B). A total of 899 (41%) participants reported both local and systemic adverse reactions (Figure S2C). 

### Association of anxiety and depression with adverse reactions

**Fig. 1:**
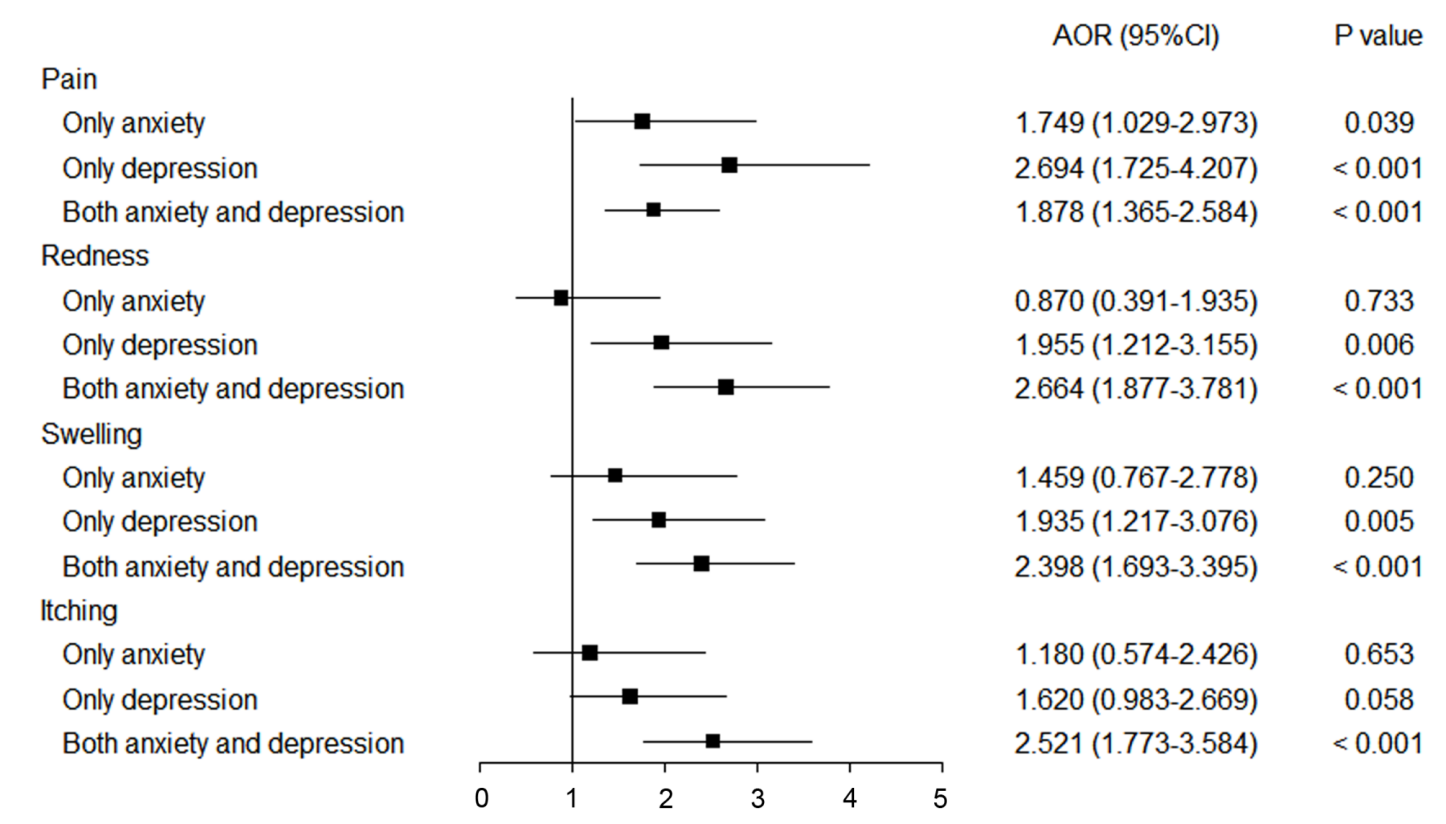
Association between anxiety, depression and self-reported local adverse reactions Reference group: neither anxiety nor depression. AOR, adjusted odds ratio; CI, confidence interval.

**Fig. 2:**
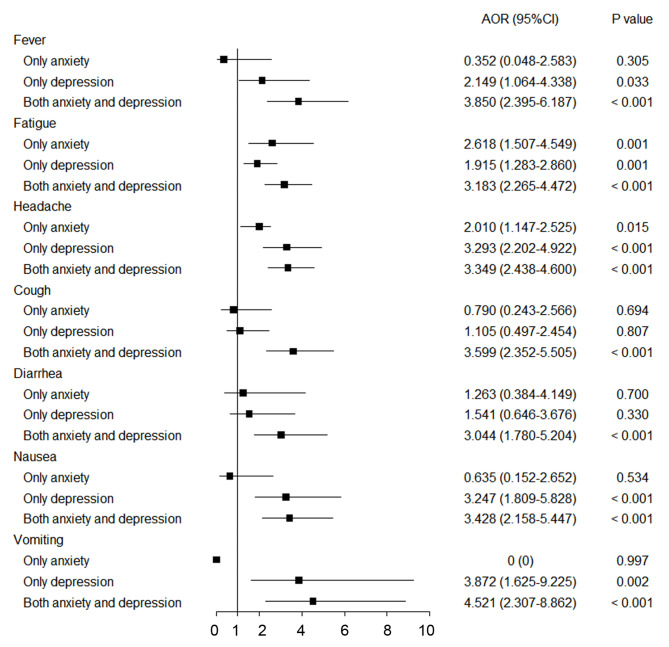
Association between anxiety, depression and self-reported systemic adverse reactions Reference group: neither anxiety nor depression. AOR, adjusted odds ratio; CI, confidence interval.

Sex, age, educational level, working condition and marital status were integrated into the multivariate logistic regression model. The results suggested that mild and moderate and above anxiety (mild: adjusted odds ratio (AOR), 3.47; 95%CI, 2.13–5.65; *P* < 0.001; moderate and above: AOR, 2.61; 95%CI, 1.21–5.62; *P* < 0.05) and depression (mild: AOR, 3.09; 95%CI, 2.01–4.77; *P* < 0.001; moderate and above: AOR, 2.95; 95%CI, 1.45–6.04; *P* < 0.01) increased the risk of overall self-reported adverse reactions, compared with without anxiety or depression (Table S2). Furthermore, anxiety (AOR, 3.80; 95%CI, 1.62–8.92; *P* < 0.01) or depression (AOR, 2.97; 95%CI, 1.64–5.39; *P* < 0.001) or both (AOR, 3.29; 95%CI, 2.06–5.26; *P* < 0.001) had increased risks of overall adverse reactions compared with neither anxiety nor depression (Table S3).Compared with those without anxiety or depression, participants with mild and moderate and above anxiety (mild: Pain: AOR, 1.71; 95%CI, 1.25–2.34; *P* < 0.01; Redness: AOR, 1.95; 95%CI, 1.36–2.79; *P* < 0.001; Swelling: AOR, 1.98; 95%CI, 1.40–2.79; *P* < 0.001; Itching: AOR, 1.94; 95%CI, 1.35–2.79; *P* < 0.001; moderate and above: Pain: AOR, 1.82; 95%CI, 1.03–3.21; *P* < 0.05; Redness: AOR, 2.29 ; 95%CI, 1.23–4.28; *P* < 0.01; Swelling: AOR, 2.19; 95%CI, 1.18–4.08; *P* < 0.05; Itching: AOR, 2.55; 95%CI, 1.40–4.64; *P* < 0.01) and depression (mild: Pain: AOR, 2.29; 95%CI, 1.69–3.12; *P* < 0.001; Redness: AOR, 2.26; 95%CI, 1.61–3.16; *P* < 0.001; Swelling: AOR, 1.83; 95%CI, 1.30–2.57; *P* < 0.001; Itching: AOR, 1.85; 95%CI, 1.30–2.62; *P* < 0.01; moderate and above: Pain: AOR, 1.58; 95%CI, 0.97–2.57; *P* > 0.05; Redness: AOR, 2.95; 95%CI, 1.74–4.99; *P* < 0.001; Swelling: AOR, 3.59; 95%CI, 2.18–5.90; *P* < 0.001; Itching: AOR, 3.30 ; 95%CI, 1.98–5.50; *P* < 0.001) were more likely to report local adverse reactions (Table 2). Besides, participants with mild and moderate and above anxiety (mild: Fever: AOR, 2.65; 95%CI, 1.61–4.35; *P* < 0.001; Fatigue: AOR, 3.39; 95%CI, 2.40–4.78; *P* < 0.001; Headache: AOR, 2.58; 95%CI, 1.89–3.52; *P* < 0.001; Cough: AOR, 3.08; 95%CI, 2.01–4.73; *P* < 0.001; Diarrhea: AOR, 2.41; 95%CI, 1.40–4.17; *P* < 0.01; Nausea: AOR, 1.90; 95%CI, 1.15–3.14; *P* < 0.05; Vomiting: AOR, 2.39; 95%CI, 1.15–4.94; *P* < 0.05; moderate and above: (Fever: AOR, 2.67; 95%CI, 1.10–6.47; *P* < 0.05; Fatigue: AOR, 1.81; 95%CI, 1.05–3.14; *P* < 0.05; Headache: AOR, 2.88; 95%CI, 1.64–5.05; *P* < 0.001; Cough: AOR, 1.72; 95%CI, 0.67–4.42; *P* > 0.05; Diarrhea: AOR, 2.74; 95%CI, 1.05–7.16; *P* < 0.05; Nausea: AOR, 4.24; 95%CI, 2.05–8.77; *P* < 0.001; Vomiting: AOR, 4.34; 95%CI, 1.47–12.86; *P* < 0.01) and depression (mild: Fever: AOR, 3.00; 95%CI, 1.87–4.83; *P* < 0.001; Fatigue: AOR, 2.61; 95%CI, 1.92–3.53; *P* < 0.001; Headache: AOR, 3.40; 95%CI, 2.54–4.55; *P* < 0.001; Cough: AOR, 2.44; 95%CI, 1.57–3.79; *P* < 0.001; Diarrhea: AOR, 2.07; 95%CI, 1.18–3.64; *P* < 0.05; Nausea: AOR, 3.35; 95%CI, 2.17–5.17; *P* < 0.001; Vomiting: AOR, 4.06; 95%CI, 2.11–7.83; *P* < 0.001; moderate and above: Fever: AOR, 4.30; 95%CI, 2.15–8.60; *P* < 0.001; Fatigue: AOR, 2.30; 95%CI, 1.39–3.79; *P* < 0.01; Headache: AOR, 2.66; 95%CI, 1.62–4.38; *P* < 0.001; Cough: AOR, 3.37; 95%CI, 1.75–6.51; *P* < 0.001; Diarrhea: AOR, 3.84; 95%CI, 1.81–8.16; *P* < 0.01; Nausea: AOR, 3.66; 95%CI, 1.83–7.30; *P* < 0.001; Vomiting: AOR, 6.09; 95%CI, 2.38–15.60; *P* < 0.001) were also more likely to report systemic adverse reactions compared with those without anxiety or depression (Table 3). Noteworthily, compared with those with neither anxiety nor depression, participants with both anxiety and depression were more subject to local (Pain: AOR, 1.88; 95%CI, 1.37–2.58; *P* < 0.001; Redness: AOR, 2.66 ; 95%CI, 1.88–3.78; *P* < 0.001; Swelling: AOR, 2.40; 95%CI, 1.69–3.40; *P* < 0.001; Itching: AOR, 2.52; 95%CI, 1.77–3.58; *P* < 0.001) and systemic (Fever: AOR, 3.85; 95%CI, 2.40–6.19; *P* < 0.001; Fatigue: AOR, 3.18; 95%CI, 2.27–4.47; *P* < 0.001; Headache: AOR, 3.35; 95%CI, 2.44–4.60; *P* < 0.001; Cough: AOR, 3.60; 95%CI, 2.35–5.51; *P* < 0.001; Diarrhea: AOR, 3.04; 95%CI, 1.78–5.20; *P* < 0.001; Nausea: AOR, 3.43; 95%CI, 2.16–5.45; *P* < 0.001; Vomiting: AOR, 4.52; 95%CI, 2.31–8.86; *P* < 0.001) adverse reactions (Figs. 1 and 2).

**Table 2:** Association between the levels of anxiety and depression and self-reported local adverse reactions

	Local adverse reactions [AOR (95%CI)]			
	Pain	Redness	Swelling	Itching
Anxiety				
Mild	1.711 (1.251–2.338) **	1.950 (1.362–2.793) ***	1.976 (1.397–2.793) ***	1.941 (1.351–2.788) ***
Moderate and above	1.822 (1.034–3.213) *	2.294 (1.230–4.278) **	2.191 (1.177–4.080) *	2.548 (1.400–4.640) **
Depression				
Mild	2.293 (1.686–3.120) ***	2.255 (1.610–3.158) ***	1.830 (1.303–2.568) ***	1.849 (1.304–2.622) **
Moderate and above	1.579 (0.971–2.569)	2.946 (1.738–4.991) ***	3.585 (2.181–5.895) ***	3.302 (1.984–5.497) ***

* *P* < 0.05; ** *P* < 0.01; *** *P* < 0.001. Reference group: without anxiety or depression. AOR, adjusted odds ratio; CI, confidence interval.

**Table 3:** Association between the levels of anxiety and depression and self-reported systemic adverse reactions

Systemic adverse reactions [AOR (95%CI)]	Anxiety			Depression	
	Mild	Moderate and above		Mild	Moderate and above
Fever	2.648 (1.613–4.348) ***	2.669 (1.102–6.466) *		3.002 (1.866–4.831) ***	4.297 (2.147–8.597) ***
Fatigue	3.391 (2.403–4.784) ***	1.813 (1.048–3.139) *		2.606 (1.924–3.531) ***	2.295 (1.390–3.788) **
Headache	2.577 (1.886–3.522) ***	2.878 (1.641–5.047) ***		3.397 (2.536–4.550) ***	2.660 (1.615–4.381) ***
Cough	3.083 (2.011–4.725) ***	1.720 (0.669–4.423)		2.442 (1.572–3.792) ***	3.373 (1.747–6.513) ***
Diarrhea	2.412 (1.395–4.170) **	2.744 (1.052–7.159) *		2.074 (1.183–3.636) *	3.843 (1.810–8.157) **
Nausea	1.899 (1.148–3.140)*	4.236 (2.046–8.769) ***		3.345 (2.165–5.168) ***	3.658 (1.833–7.303) ***
Vomiting	2.387 (1.153–4.941) *	4.342 (1.467–12.858)**		4.059 (2.105–7.825) ***	6.089 (2.376–15.604) ***

* *P* < 0.05; ** *P* < 0.01; *** *P* < 0.001. Reference group: without anxiety or depression. AOR, adjusted odds ratio; CI, confidence interval.

## Discussion

Individuals’ hesitancy to receive COVID-19 vaccine is mainly attributed to vaccine safety [42, 43]. Therefore, identifying factors amplifying adverse reactions of the vaccine is a concern. It will be helpful to promote vaccination, achieve herd immunity and stop the spread of the COVID-19 pandemic. During the COVID-19 pandemic, anxiety and depression are more prevalent [44, 45]. Considering this situation, we explored the effect of anxiety and depression on safety of COVID-19 vaccine recipients. The results mainly found that anxiety and depression significantly increased the risk of self-reported local and systemic adverse reactions (*P* < 0.05). Moreover, participants with moderate and above anxiety or depression and with both anxiety and depression were more likely to report local and systemic adverse reactions (*P* < 0.05).

In our study, the prevalence of anxiety and depression was 13% and 15%, respectively. These results are generally lower than in prior studies: a meta-analysis showed that the prevalence of depression and anxiety was 26.9% and 21.8%, respectively, during the COVID-19 epidemic in China [46]; a study from 63 countries found that 59% of participants reported anxiety symptoms and 39% reported depressive symptoms [47]; and another study reported that 50.3% and 41.3% of participants from an urban, low-income public university were identified with depression and anxiety, respectively.^20^ After the COVID-19 outbreak, the National Health Commission of China immediately releases the guidelines for emergency psychological crisis intervention for the COVID-19 pandemic for different populations [48]. 24-hour mental hotlines are quickly established across China to provide the public and healthcare workers with psychological counseling. Moreover, the mental health handbook and social media (e.g., Weibo, WeChat, etc.) are utilized to share strategies and guidelines dealing with psychological distress [48]. Therefore, lower anxiety and depression rates are inseparable from rapid national guidelines for emergency psychological crisis intervention, as well as timely, comprehensive and precise prevention and control measures of Shanghai’s government [49].

The results showed that 74% of participants reported at least one adverse reaction and all adverse reactions were mild or moderate in severity. The most commonly reported local adverse reaction was pain at the injection site and systemic adverse reactions were fatigue and headache. Previous multiple clinical randomized controlled trials of vaccines reported that most adverse reactions were mild or moderate, with the most common local adverse reaction being pain at the injection site, and the most common systemic adverse reactions being fatigue, headache and fever (vaccines included adenovirus vector vaccine, mRNA vaccine, protein subunit vaccine and inactivated virus vaccine) [12, 15–17, 21, 22, 50, 51]. All participants in our study received inactivated COVID-19 vaccine, but the incidence of reported adverse reactions was higher than other published clinical trials of inactivated COVID-19 vaccines (Xia et al., 29%; Zhang et al., 26%; Wu et al., 21%; Han et al., 27%; Ella et al., 21%) [12, 14–16, 52]. Younger people may be more likely to report adverse reactions than older people [15, 16, 18, 21, 53]. A cohort study among19 586 adults receiving a COVID-19 vaccination indicated that younger age had an increased risk of adverse reactions [54]. 90% of participants were aged 18–40 years and 63% were 18–25 years in our study, and this may be related to the relatively high incidence of self-reported adverse reactions in the present study.

Our study suggested that participants with anxiety or depression or both were more likely to report local and systemic adverse reactions compared with those without anxiety or depression, consistent with previous findings. A study also reported that COVID-19 vaccine associated anxiety increased reported adverse reactions to vaccination in patients with chronic liver disease [55]. Similarly, a Japanese study suggested that psychological distress was positively associated with adverse reactions after COVID-19 vaccination [56]. Vaccine enters the body and immediately activate the immune system, and inflammatory cytokines are released from macrophages due to early innate immune response [28]. Anxiety or depression causes an increased level of Interleukin-6 in serum [57, 58], which can enhance the immune response to vaccine and induce adverse reactions [59, 60].

There are some implications in our study. Alleviating people’s anxiety or depression related to COVID-19 or SARS-CoV-2 vaccine can help reduce or mitigate adverse reactions, in turn, that can facilitate smoother vaccination processes. Therefore, it is necessary to provide up-to-date and accurate information about COVID-19 and SARS-CoV-2 vaccine to improve the confidence of the public to SARS-CoV-2 vaccine [27]. Besides, providing services in assessing psychological status and targeted psychological interventions is crucial. Psychological interventions, including meditation/mindfulness, massage, expressive writing and cognitive behavioral stress management, can be effective vaccine adjuvants to boost vaccine effectiveness [30]. In addition, a short bout of pre-vaccination exercise can decrease reports of local and systemic adverse reactions [61]. Further work in prospective studies of psychological interventions is needed to find effective psychological intervention measures to mitigate anxiety and depression in the general population.

To our knowledge, this is the first cross-sectional study to evaluate the effect of anxiety and depression on adverse reactions to COVID-19 vaccination among the general population in China, but it still has some limitations. Firstly, data are collected based on self-reports of participants, thus, recall bias is unavoidable. Secondly, participants are only from Shanghai and all receive inactivated COVID-19 vaccine, so the results lack generalizability for other vaccines and regional populations. Thirdly, we only collect demographic data including age, sex, educational level, residential address, working condition and marital status, the interpretation for the findings may be influenced by other confounding factors. Eventually, the cross-sectional study is difficult to provide causal evidence of anxiety, depression and adverse reactions. Therefore, the findings should be interpreted with caution and more prospective studies with large samples need to be carried out to investigate their causal relationship.

## Conclusion

Anxiety and depression increased the risk of self-reported local and systemic adverse reactions to COVID-19 vaccine. Thus, assessing psychological status and appropriate psychological interventions before vaccination will help reduce or alleviate adverse reactions associated with vaccination. Furtherly, more prospective studies with large samples assessing the causal association between anxiety, depression and adverse reactions of COVID-19 vaccine are warranted.

## Electronic supplementary material

Below is the link to the electronic supplementary material.

Supplementary Material 1

## Data Availability

The data of this study is available from the corresponding author on reasonable request.
