# Supplementary material for: Effect of anxiety and depression on self-reported adverse reactions to COVID-19 vaccine: a cross-sectional study in Shanghai, China
Source: BMC Public Health. 2023 Mar 3;23:425. doi: 10.1186/s12889-023-15118-8 (PMC9983539; doi:10.1186/s12889-023-15118-8)
Supplement: Supplementary file 1 — Supplementary Material 1 [file 12889_2023_15118_MOESM1_ESM.docx]

**
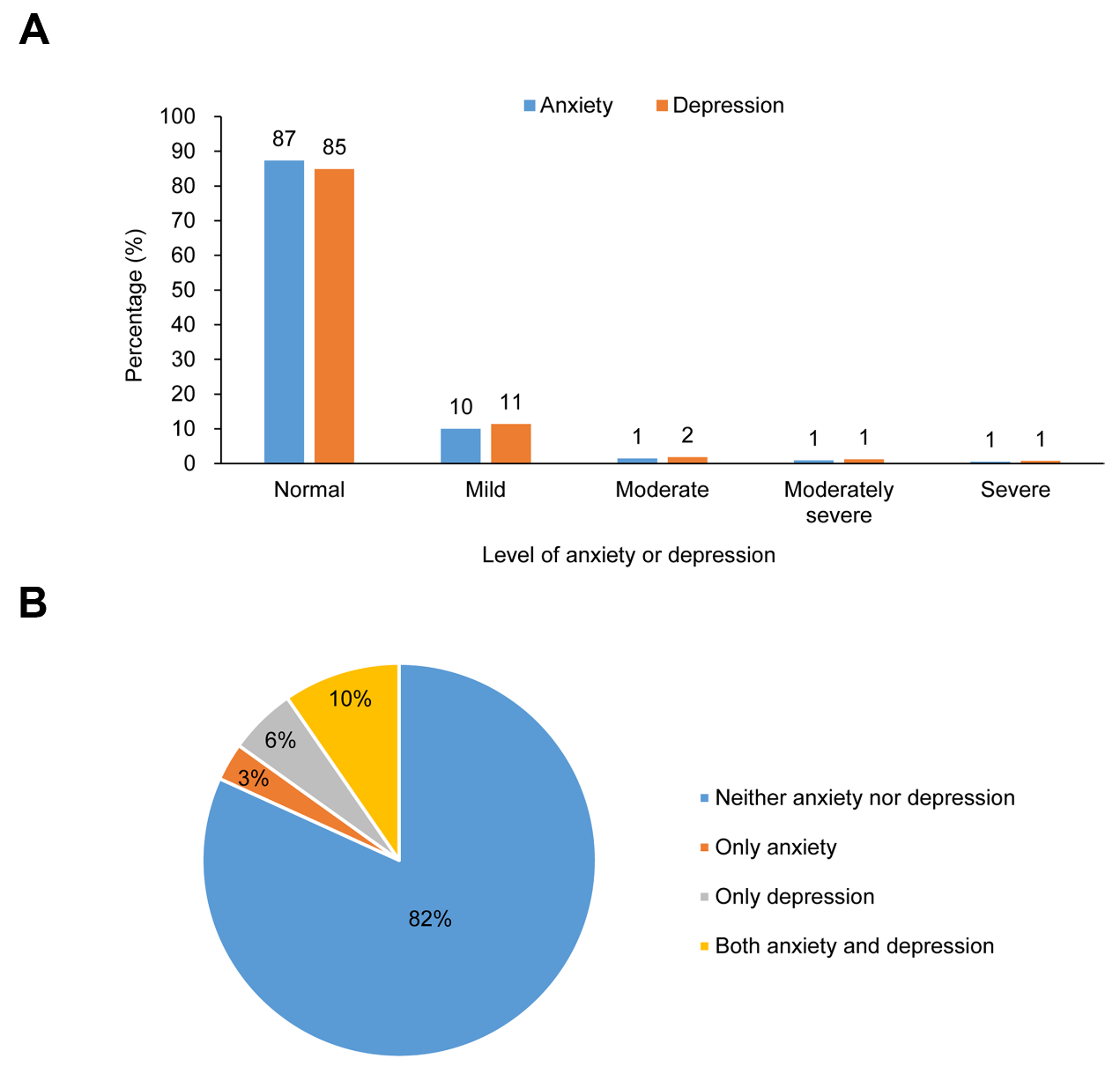
**

**Figure S1.** **Prevalence of anxiety and depression**

**
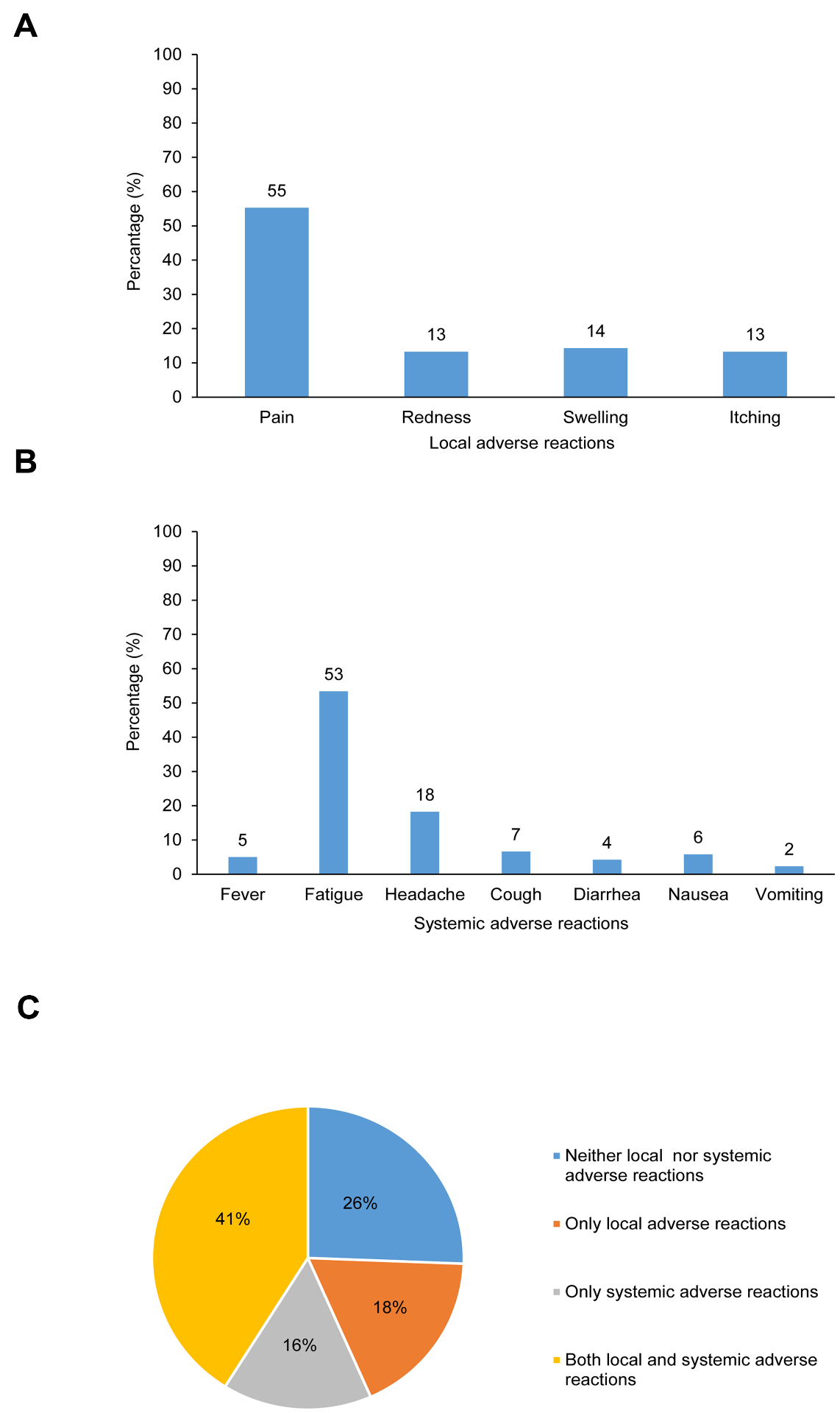
**

**Figure S2. Incidence of self-reported local and systemic adverse reactions**

**Table S1. Grades of adverse reactions**

| **Adverse reactions** | **Grade 1** | **Grade 2** | **Grade 3** | **Grade 4** |
| --- | --- | --- | --- | --- |
| **Local adverse reactions ( injection site)** |  |  |  |  |
| Pain | Does not interfere or slightly interferes with body activity | Interferes with body activity | Interferes with daily activity | Emergency or hospitalization |
| Redness ( diameter) | 2.5 ~＜5 cm and does not interfere or slightly interferes with daily activity | 5～＜10 cm or interferes with daily activity | ≥10 cm in diameter or ulceration or secondary infection or phlebitis or aseptic abscess or wound drainage or seriously interferes with daily activity | Abscesses, exfoliative dermatitis, dermal or deep tissue necrosis |
| Swelling ( diameter) | 2.5 ~＜5 cm or does not interfere or slightly interferes with daily activity | 5～＜10 cm or interferes with daily activity | ≥10 cm in diameter or ulceration or secondary infection or phlebitis or aseptic abscess or wound drainage or seriously interferes with daily activity | Abscesses, exfoliative dermatitis, dermal or deep tissue necrosis |
| Itching | Pruritus at the inoculation site was relieved by itself or within 48 hours after treatment | Pruritus at the inoculation site was not relieved within 48h after treatment | Interferes with daily activity | NA |
| **Systemic adverse reactions** |  |  |  |  |
| Fever  (axillary temperature) | 37.3～＜38.0 ℃ | 38.0～＜38.5℃ | 38.5～＜39.5℃ | ≥ 39.5℃ and lasts more than 3 days |
| Fatigue | Does not interfere with daily activity | Interferes with daily activity | Seriously interferes with daily activity and unable to work | Emergency or hospitalization |
| Headache | Does not interfere with daily activity, does not require treatment | Mildly interferes with daily activity, may require treatment or intervention | Seriously interferes with daily activity, requires treatment or intervention | Emergency or Hospitalization |
| Cough | Transient and does not require treatment | Persist and treatment works | Sporadic and treatment does not control | Emergency or Hospitalization |
| Diarrhea | 3～4 times / 24 hours | 5～7 times / 24 hours | > 7 times / 24 hours or require intravenous fluids > 2L | Emergency or Hospitalization |
| Nausea | Transient (< 24 hours) | Persistent (24 ~ 48 hours) | Persistent (> 48 hours) or requires intravenous fluids > 2L | Emergency or Hospitalization |
| Vomiting | 1~2 times / 24 hours and does not interfere with daily activity | 3～5times / 24 hours or activity is restricted | > 6 times / 24 hours or requires intravenous fluids | Emergency or Hospitalization |

**Table S2. Association between the levels of anxiety and depression and overall self-reported adverse reactions**

|  | Overall adverse reactions  [AOR (95%CI)] | Overall local adverse reactions [AOR (95%CI)] | Overall systemic adverse reactions [AOR (95%CI)] |
| --- | --- | --- | --- |
| Anxiety |  |  |  |
| Mild | 3.472 (2.133-5.652)*** | 1.884 (1.359-2.611)*** | 3.850 (2.663-5.566)*** |
| Moderate and above | 2.607 (1.209-5.622)* | 1.982 (1.100-3.572)* | 2.006 (1.135-3.545)* |
| Depression |  |  |  |
| Mild | 3.094 (2.007-4.768)*** | 2.493 (1.803-3.445)*** | 3.055 (2.211-4.221)*** |
| Moderate and above | 2.954 (1.445-6.040)** | 1.716 (1.037-2.840)* | 2.470 (1.465-4.465)** |

* *P* < 0.05; ** *P* < 0.01; *** *P* < 0.001. Reference group: without anxiety or depression. AOR, adjusted odds ratio; CI, confidence interval.

**Table S3. Association between anxiety, depression and overall self-reported adverse reactions**

|  | Overall adverse reactions [AOR (95%CI)] | Overall local adverse reactions [AOR (95%CI)] | Overall systemic adverse reactions [AOR (95%CI)] |
| --- | --- | --- | --- |
| Only anxiety | 3.801 (1.620-8.916)** | 1.626 (0.951-2.780) | 3.016 (1.679-5.418)*** |
| Only depression | 2.972 (1.639-5.388)*** | 2.550 (1.616-4.023)*** | 2.280 (1.495-3.477)*** |
| Both anxiety and depression | 3.291 (2.057-5.264)*** | 2.175 (1.553-3.046)*** | 3.588 (2.501-5.148)*** |

* *P* < 0.05; ** *P* < 0.01; *** *P* < 0.001. Reference group: neither anxiety nor depression. AOR, adjusted odds ratio; CI, confidence interval.
